# Dynamic Fracture Behavior of Steel Fiber Reinforced Self-Compacting Concretes (SFRSCCs)

**DOI:** 10.3390/ma10111270

**Published:** 2017-11-05

**Authors:** Xiaoxin Zhang, Gonzalo Ruiz, Manuel Tarifa, David Cendón, Francisco Gálvez, Waleed H. Alhazmi

**Affiliations:** 1 ETSI de Caminos, Canales y Puertos, Universidad de Castilla-La Mancha, 13071 Ciudad Real, Spain; gonzalo.ruiz@uclm.es (G.R.); manuelagustin.tarifa@uclm.es (M.T.); 2 ETSI de Caminos, Canales y Puertos, Universidad Politécnica de Madrid, 28040 Madrid, Spain; dcendon@mater.upm.es (D.C.); fgalvez@mater.upm.es (F.G.); waleed@mater.upm.es (W.H.A.)

**Keywords:** steel fiber reinforced self-compacting concrete (SFRSCC), fiber content, drop-weight impact test, flexure, dynamic increase factor (DIF)

## Abstract

Three-point bending tests on notched beams of three types of steel fiber-reinforced self-compacting concrete (SFRSCC) have been performed by using both a servo-hydraulic machine and a drop-weight impact instrument. The lo ading rates had a range of six orders of magnitude from 2.20 × 10^−3^ mm/s (quasi-static) to 2.66 × 10^3^ mm/s. These SFRSCCs had the same matrix, but various types of steel fiber (straight and hooked-end) and contents (volume ratios), 0.51%, 0.77% and 1.23%, respectively. The results demonstrate that the fracture energy and the flexural strength increase as the loading rate increases. Moreover, such tendency is relatively moderate at low rates. However, at high rates it is accentuated. For the 0.51% fiber content, the dynamic increase factors of the flexural strength and the fracture energy are approximately 6 and 3, while for the 1.23% fiber content, they are around 4 and 2, respectively. Thus, the higher the fiber content the less rate sensitivity there is.

## 1. Introduction

Since self-compacting concrete (SCC) was developed in 1988 [1], it has been widely used in the construction industry due to the fact that the placement and quality control of SCC are much easier than those of conventional vibrated concrete (CVC) because of its characteristics of super fluidity and self-consolidation. However, SCC has higher content in fine aggregates, which results in shorter characteristic length, less toughness and more brittleness than CVC with similar compressive strength [2,3,4]. This makes SCC structures vulnerable to impulsive loads, such as beam-column joints and coupling beams subject to earthquake loading, in which the strain rate could reach values up to 1/s [5]. Thus, a feasible solution is to use steel or other types of fiber to improve the toughness and ductility of SCC. The mix of steel fibers and SCC is commonly referred to as steel fiber-reinforced SCC (SFRSCC). 

Until the present time, most studies have focused on the quasi-static mechanical behavior of SCC and SFRSCC [6,7,8,9,10,11,12,13,14,15,16,17,18]. However, there has been limited research on the impact behavior of SFRSCC [19,20,21,22] compared with the studies conducted on conventional vibrated steel fiber-reinforced concrete [23,24,25,26,27,28,29]. At times, in order to obtain better fluidity, coarse aggregate is removed from SFRSCC. Subsequently, it may also be considered a type of high-performance fiber-reinforced cementitious composite (HPFRCC), which is characterized by high ductility and tension-hardening behavior in statics [30,31]. Currently, the dynamic behavior of this material is becoming more of a concern as well. Recently, Caverzan, di Prisco and Cadoni [32] have studied the influence of fiber dispersion on the dynamic behavior of HPFRCC by using a hydro-pneumatic machine and a modified Hopkinson bar. They observed that under a dynamic regime, the influence of fiber distribution (random or aligned distributed fibers) on peak strength was reduced. While in static conditions, aligned fibers favored stable crack propagation. Habel and Gauvreau [33] have used a drop weight impact machine to study the dynamic fracture behavior of a SFRSCC. Straight steel fibers (length/diameter: 10 mm/0.2 mm), 430 kg/m^3^, were added to the self-consolidating mix and 10.3 kg and 20.6 kg hammers were used for the impact tests, corresponding to the loading rates of 4.2 m/s and 4.3 m/s, respectively. The results show that the bending resistance increased by more than 25% relative to the static resistance. Caverzan, Cadoni and Di Prisco [20] have used a modified Hopkinson bar (MHB) to perform the tensile tests on SFRSCC. The hooked-end steel fiber content was 50 kg/m^3^ with a fiber length of 35 mm and a diameter of 0.5 mm. It was observed that under the displacement rate of 1.2 m/s, the dynamic increase factor (DIF) on peak strength was 1.67. Parant and Rossi et al. [22] showed that the ultra-high performance fiber-reinforced concrete with a steel fiber content of 11% had a greater strain rate sensitivity compared to the cement-based matrix and other steel fiber-reinforced concretes (fiber content less than 3%). However, the matrix of ultra-high performance fiber-reinforced concrete was modified in order to maintain workability, that is, replacing the volume of the sand as with steel fibers. Moreover, the modulus of rupture increased by 25% within the range of quasi-static loading rates (1.25 × 10^−4^ to 1.25 GPa/s) and quadrupled for loading rates greater than 500 GPa/s. Nevertheless, experimental data on the rate sensitivity and the influence of fiber content on the dynamic fracture behavior of SFRSCC are still scarce. Thus, in order to gain additional insight into the rate effect of the mechanical properties of SFRSCC and especially in fracture energy, in the paper, the dynamic fracture behavior of three different SFRSCCs is presented. The results show that the fracture energy and the flexural strength increase as the loading rate increases for these three SFRSCCs. Moreover, such a tendency is relatively slight at low rates, while it is more evident at high loading rates. Furthermore, the higher the fiber content the less rate sensitivity there is.

The remainder of the paper is structured as follows: the experimental procedure is described in Section 2; the results are presented and discussed in Section 3. Finally, our conclusions are set forth in Section 4.

## 2. Experimental Procedure

Three different SFRSCCs were provided by the Spanish company PACADAR (Madrid, Spain), all of which had the same matrix, but different fiber types and contents. These three concretes are variations of a concrete mix commercially used in critical infrastructures characterized by structural members with high ratios of slenderness. A series of specimens (cylinders and notched beams) were fabricated for the experiment. Three-point bending tests on notched beams were performed at a loading rate which ranged from 10^−3^ mm/s to 10^3^ mm/s, corresponding to notch root strain rates from 10^−5^ s^−1^ to 10^1^ s^−1^. Both a servo-hydraulic testing machine and a drop-weight impact instrument were used. Four specimens were tested at each loading condition. Moreover, in order to eliminate age effect on the properties, all tests were performed at around seven months. 

### 2.1. Material Characterization

In general, for fiber-reinforced concrete, fibers provide post-cracking ductility. When the load is less than the first crack load in load-displacement (deflection) curves, it is called deflection-softening behavior [24,30,34]. The contrary situation is referred to as deflection-hardening. In our case, three types of SFRSCC designed and fabricated by PACADAR following the authors’ requirements. Namely, they should share the same matrix while having different post-cracking behaviors. Deflection-softening behavior was labeled as Concrete PA, mild and marked deflection-hardening behaviors for Concrete PB and Concrete PC, respectively. Their matrices were kept constant as required. Two different sands, Sand I (0–0.8 mm) (INCUSA, Segovia, Spain) and Sand II (0–2 mm) (INCUSA, Segovia, Spain), CEM I cement 42.5 R-SR (Cementos La Unión, Valencia, Spain) and two types of superplasticizer (Glenium ACE-325 and B-225, BASF, Barcelona, Spain) were used in the composition. The mixing proportions by weight were: 1:0.12:0.35:1.21:1.27:0.38:0.021 (cement: silica fume: filler siliceous: Sand I: Sand II: water: superplasticizer). Straight smooth short steel fibers and hooked-end long steel fibers were added as reinforcement. The former (Dramix OL 13/0.20) (BEKAERT, Burgos, Spain) were 13 mm in length, 0.20 mm in diameter and 65 in aspect ratio, the minimum tensile strength is 2600 MPa. The latter (Dramix RC 80/30 BP) (BEKAERT, Burgos, Spain) were 30 mm in length, 0.38 mm in diameter and 80 in aspect ratio, with a minimum tensile strength of 1050 MPa. The quantities and fiber type used in each concrete are shown in Table 1.

**Figure 1:**
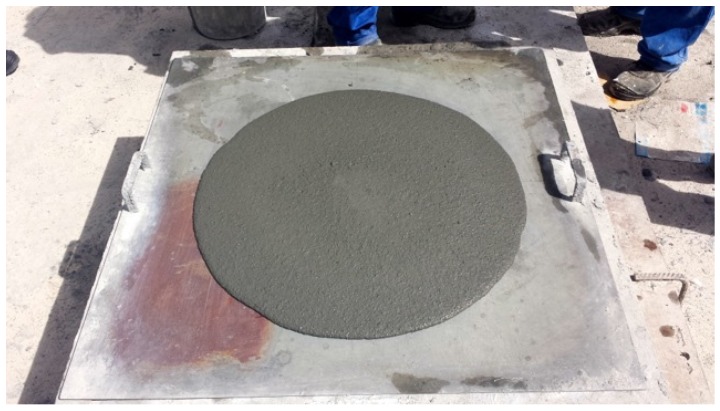
Slump flow test result (Concrete PC).

The workability of the fresh mixtures was determined following the ASTM C1611 standard [35]. In this sense, all mixes showed consistent and homogenous slump flow test results as shown in Figure 1. The slump flow values are listed in Table 1. It is observed that increasing fiber content make the fluidity of the mixtures difficult. Thus, the workability decreases [36], that is, the diameter of the slump flow test decreases.

In order to eliminate age effect on the strength, compressive tests were conducted by a servo-hydraulic testing machine following ASTM C39 [37] and ASTM 469 [38] standards at an age approximately seven months, which is similar to the age of beams tested under three-point bending. Four cylinders of 150 mm × 300 mm (diameter × height) were tested for each type of SFRSCC. The results are presented in Table 2. It is obvious that the compressive strength and the elastic modulus are almost unaffected by the fiber content [39].

### 2.2. Three-Point Bending Tests

Three-point bending tests on notched beams at a wide range of loading rates, from 10^−3^ mm/s to 10^3^ mm/s, were performed by a servo-hydraulic testing machine and a drop-weight impact instrument.

**Figure 2:**
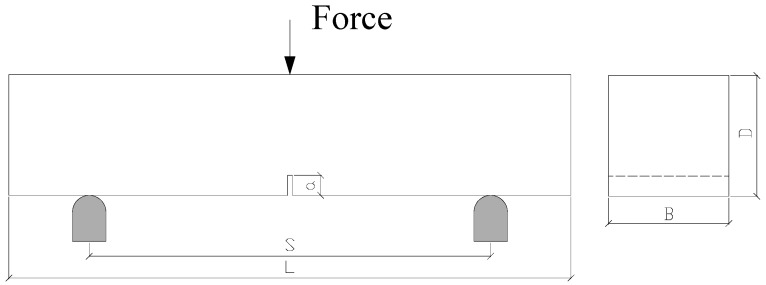
Schematic diagram of the specimen.

The dimensions of the beams were 100 mm × 100 mm (B × D) mm in cross-section, and 450 mm in total length (L). The span (S) was set at 333 mm during the tests and, the initial notch-depth ratio (a/D) was approximately 1/6, see Figure 2. Accordingly, the recommendation of the RILEM TC 162-TDF committee [40] and the EN 14651 standard [41] was followed with a reduction factor of 1.5, due to the fact that only these were the molds available at the factory.

#### 2.2.1. Tests under Low Loading Rates Ranging from 10^−3^ to 10^1^ mm/s

**Figure 3:**
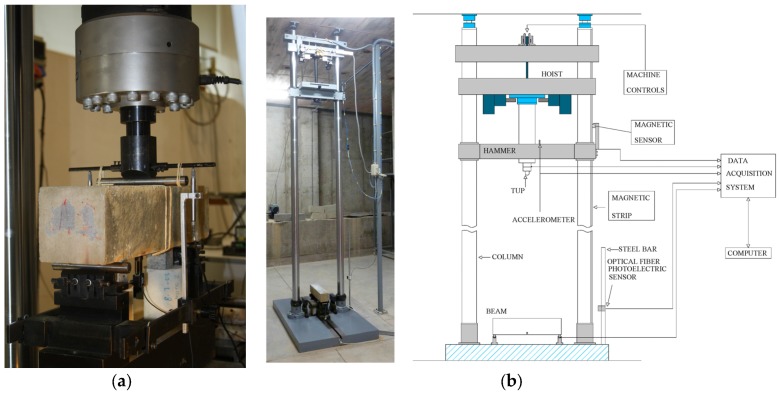
(**a**) Photo of the servo-hydraulic testing machine; (**b**) photo and schematic diagram of the drop-weight impact machine.

For this low loading rate range, the tests were performed by using the servo-hydraulic testing machine, as shown in Figure 3a.

The beam rests on two rigid-steel cylinders placed on two supports which permit rotation out of the plane of the beam and rolling along the longitudinal axis of the beam with negligible friction. These supports roll on the upper surface of a very stiff beam fastened to the machine base. Two LVDTs (linear variable differential transducers) fixed to the steel beam are used to measure the displacement between the loading rod and the steel beam. The deformation of the supports can be ignored due to their great stiffness during the test. Moreover, an extensometer attached to the lower surface of the beam was used to obtain the crack-mouth opening displacement (CMOD). The tests were conducted in the position control. Two loading rates were applied during the test from a quasi-static level (2.20 × 10^−3^ mm/s) to a rate dependent level (2.20 × 10^1^ mm/s). Four specimens were tested at each loading rate.

#### 2.2.2. Impact Tests at a Loading Rate of 10^3^ mm/s

At this high loading rate, all tests were performed by using the drop-weight impact machine as shown in Figure 3b, which consists of mechanical part and data acquisition system [42]. It has the capacity to drop a maximum mass of 316 kg from a height of up to 2.6 m and can accommodate flexural beams with spans of up to approximately 1.6 m. An impact hammer weighing 120.6 kg was used for the three-point bending tests and two drop heights of 160 mm and 360 mm were selected. The corresponding impact velocities were 1.77 × 10^3^ mm/s and 2.66 × 10^3^ mm/s, respectively.

The impact force between the hammer tup and the beam was measured by a piezoelectric force sensor affixed to the tup. Due to the fact that the impact load measured included inertia force, thus, two additional force sensors were placed between the supporter and the specimen in order to measure pure bending load (reaction force) excluding the inertia effect [42,43,44,45]. An accelerometer bonded to the hammer was used to measure acceleration during the impact process. The initial hammer impact velocity was defined by the drop height and the gravitational acceleration. The hammer velocity during the impact period was obtained through the initial impact velocity and the integral of the acceleration recorded by the accelerometer. Subsequently, loading point displacement was determined by the integral of the hammer velocity [25,46]. Once the reaction force and the loading point displacement were obtained, that is, the inertia effect was removed during the impact process, the dynamic fracture energy was obtained. Note that this method would no longer be valid if the failure pattern were changed to shear or local rather than flexure.

Furthermore, a three-point bending condition had to be confirmed as well during the impact process by using this method, namely, the loss of contact between the tup, the specimen and the supports were to be avoided, which was checked by a high speed video camera [47]. If there were a loss of contact among them under the impact loading condition, a wrong interpretation would result from experimental results by using the method, such as the work done in reference [42].

The fracture energy could be calculated according to Equation (1). Note that the first item in the equation represents the fracture energy externally supplied to propagate the crack across the specimen. While the second item corresponds to the energy absorbed by the beam due to the self-weight, it reduces the error to approximately 60% compared to that recommended by the RILEM TC50-FMC Technical Committee [48], which did not consider the influence of the cantilever of the beam.

(1) $$\mathrm{GF}\;=\;\frac{\mathrm{Wo}}{B\left(D-a\right)}\;+\;\frac{\mathrm{mg}\left(1-\frac{L}{2S}\right)\delta_{s}}{B\left(D-a\right)}$$

where ${\;W}_{0},\;B,\;D,\;a,\;S,\;L,\;m,{\;\delta }_{s}$ and g are the area under the experimental load-displacement curve, width, depth, notch, span, length, mass, specified deflection of the beam and gravitational acceleration, respectively. Under dynamic loading conditions, $W_{0}$ was obtained by the area under the reaction force-displacement (load–displacement) curves as in [42,43,44,45]. Moreover, the flexural strength for a notched beam with center-point loading can be calculated as Equation (2).

(2) $$R=\frac{3P_{\max}S}{2B{\left(D-a\right)}^{2}}$$

where Pmax is the peak load in the load-displacement curve.

### 2.3. Determination of Elastic Modulus by Using a Three-Point Bending Test

For span/depth (S/D) ratios (β) between 2.5 and 16, the elastic modulus obtained from prisms could be calcualted by general Equations (3) and (4) according to the reference [49].

(3) $$E=6\frac{\;\mathrm{Sa}}{C_{i}{\mathrm{BD}}^{2}}{\;\upsilon }_{\beta }\left(\alpha \right)$$

(4) $$\upsilon_{\beta }\left(\alpha \right)=\upsilon_{\beta }\left(a/D\right)=0.8-1.7\alpha +2.4\alpha^{2}+\frac{0.66}{{\left(1-\alpha \right)}^{2}}+\frac{4}{\beta }\left(-0.04-0.58\alpha +1.47\alpha^{2}-2.04\alpha^{3}\right)$$

where $C_{i}$ is the initial compliance determined from the load-CMOD curve, $\upsilon_{\beta }\left(\alpha \right)$ is a dimensionless shape function depending on β and the relative notch/depth ratio α; the other parameters of the beam were defined previously (see Figure 2). This procedure was used to determine the elastic modulus of the specimens tested at the lowest loading rate, 2.20 × 10^−3^ mm/s.

## 3. Results and Discussion

### 3.1. Failure Pattern and Fracture Surfaces

All beams tested present flexural failure pattern. For the impact tests, only the beams of Concrete PC were not broken into two halves because there was not enough impact energy for the hammer drop height of 160 mm. The remaining beams were fractured completely.

**Figure 4:**
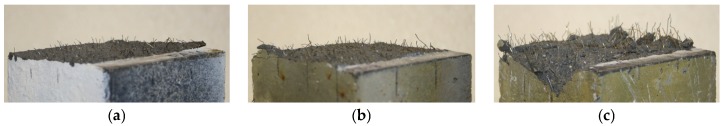
Morphology of fracture surface of different concretes at loading rate 2.66 × 10^3^ mm/s. (**a**) Concrete PA; (**b**) Concrete PB; (**c**) Concrete PC.

Figure 4 shows the morphology of fracture surface of Concretes PA, PB and PC at the impact loading rate of 2.66 × 10^3^ mm/s, respectively. It is obvious that Concrete PC exhibits greater roughness on the fracture (crack) surface than the others due to the fact that it has a higher fiber content. More fibers increase resistance to crack propagation and provide a better bridging crack effect. Moreover, no broken fibers were found in any case. The fibers in the crack surface were pulled out.

### 3.2. Quasi-Static Flexural Behavior

**Figure 5:**
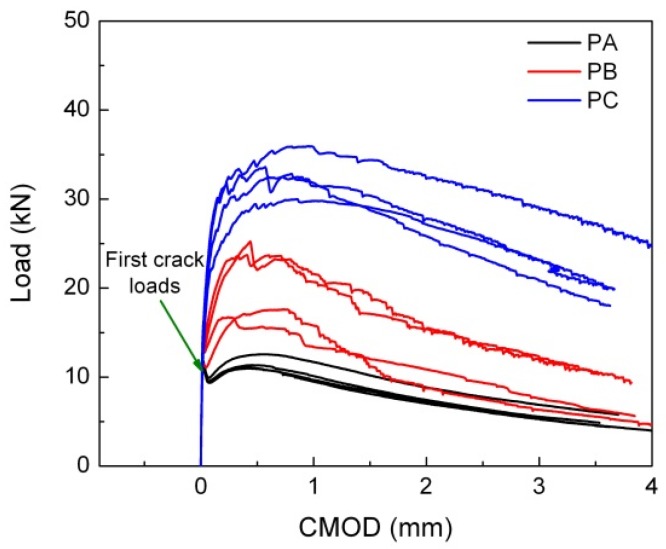
Load-crack-mouth opening displacement (CMOD) curves.

Figure 5 presents all load-CMOD curves for each SFRSCC at their quasi-static loading rate, 2.2 × 10^−3^ mm/s. It is obvious that both the ascending (pre-peak) and descending (post-peak) parts of the curves are influenced by the addition of steel fibers. For Concrete PA with only smooth fibers and the lowest fiber content of 0.51%, the behavior follows that of conventional SFRSCC. Namely, the fibers provide post-cracking ductility, but the loads are less than the first crack load (deflection-softening behavior). By adding more hooked-end fibers to Concrete PA, Concretes PB and PC (fiber contents 0.77% and 1.23% respectively) show different flexural behavior and can be classified as high-performance SFRSCC due to the fact that the fibers act to increase both the strength and toughness of the concretes (deflection-hardening behavior) [24,30,34]. Moreover, Concretes PB and PC may be considered a type of HPFRCC.

Furthermore, it is observed that peak load increases with an increase in the steel fiber volume ratio. The deflection corresponding to the peak load substantially increases as well, which is caused by the superb fiber bridging around the crack surface (fracture surface). This results in a higher load bearing capacity after first crack loads. Moreover, the residual flexural strength also increases due to more fiber interaction with the matrix at different material scales.

Table 3 compares the elastic modulus by using two different measuring methods. The first is the compressive test and the other is the three-point bending test, see Equation (3). The results coincide and the relative error is less than 6%. It is clear that it is still appropriate for obtaining elastic modulus conducting a three-point bending test for fiber-reinforced concretes.

### 3.3. Dynamic Flexural Behavior

#### 3.3.1. Load-Time and Load-Displacement Response

**Figure 6:**
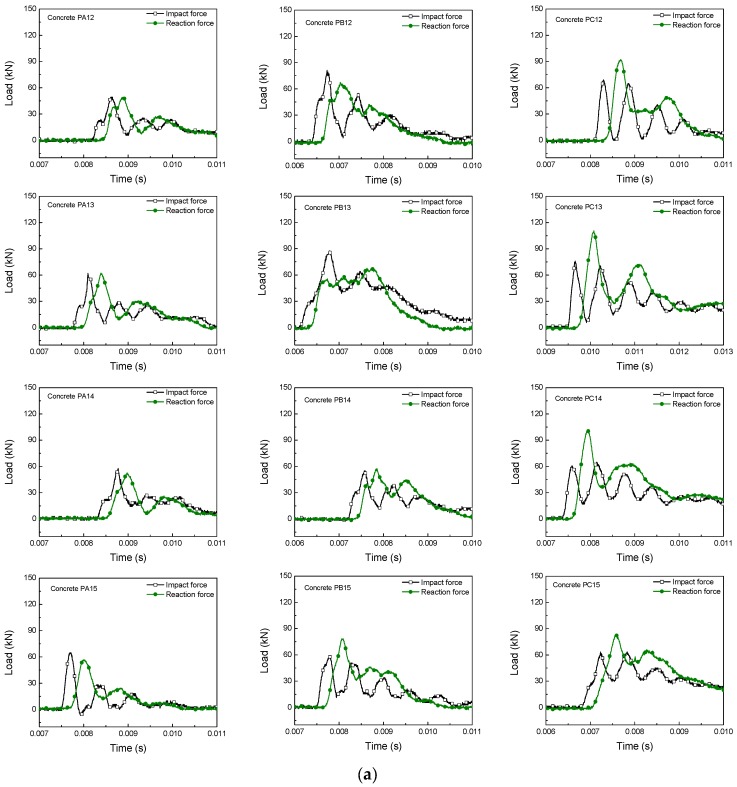

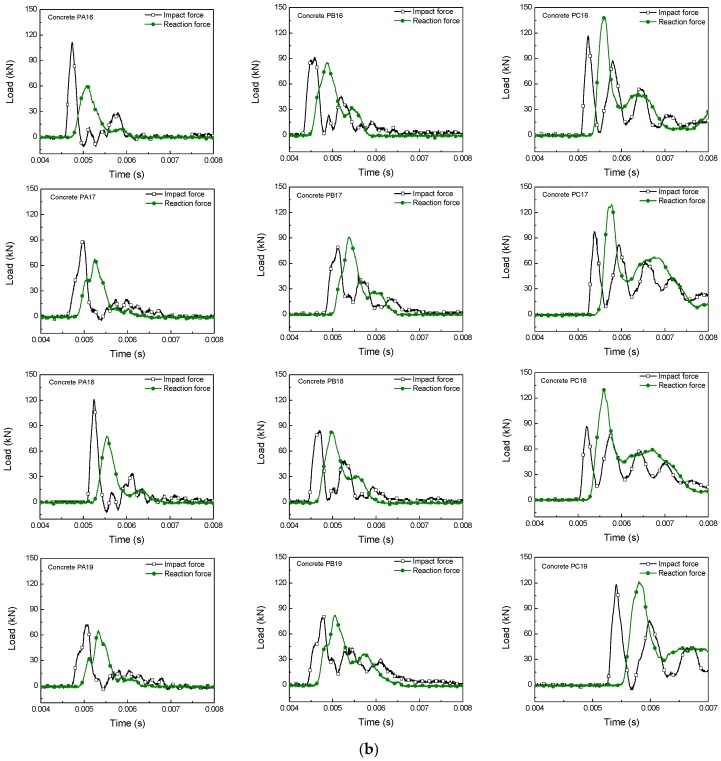
Impact and reaction forces versus time at different impact rates. (**a**) Hammer drop height 160 mm, loading rate 1.77 × 10^3^ mm/s; (**b**) Hammer drop height 360 mm, loading rate 2.66 × 10^3^ mm/s.

Figure 6 shows the impact and reaction forces versus time curves of all specimens tested in the drop-weight impact machine. There is a time delay between the impact force and the reaction in all cases. This results from, the imperfection of the contact between the support and the beam, and from another time interval of stress wave propagation from the impact point to the supports [25]. Moreover, the curves show the second peak or multiple ones due to the fiber-bridging effect, after the formation of the first cracking in the matrix. If no fiber were included in the matrix, the impact and reaction forces would decrease to zero. Furthermore, the second peak load or multiple ones increases with fiber contents due to the fact that most of the applied load is resisted by the fibers once a crack is formed.

**Figure 7:**
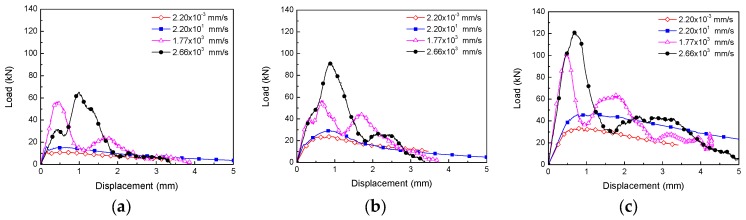
Load-displacement curves at different loading rates. (**a**) Concrete PA; (**b**) Concrete PB; (**c**) Concrete PC.

Figure 7 shows the comparison of the load-displacement (deflection) curves at different loading rates for each SFRSCC, from 10^−3^ mm/s to 10^3^ mm/s. It is worth noting that at a loading rate of 10^3^ mm/s, that is, drop-weight impact tests, the load refers to the reaction force from the supports. From the figure, it is obvious that the peak load increases with the increase in the loading rate. However, the stiffness of the beam does not present a clear tendency, which is due to the sensitivity of the elastic flexibility of the beam to the boundary conditions during the application of the concentrated load as set forth in reference [50]. Furthermore, the chosen cutoff value in displacement was set at 3 mm for calculating the fracture energy due to the fact that some beams were broken when the displacement reached approximately 3.3 mm. From now on, this fracture energy measured up to 3 mm in displacement is termed as fracture energy@3mm.

#### 3.3.2. Loading Rate Effects on DIF

Table 4 presents the experimental results at different loading rates. The dynamic increase factor (DIF) is determined by the ratios of the flexural strength (R) and the fracture energy@3mm (G_F_@3mm) to their corresponding quasi-static values for each type of SFRSCC. Herein, the lowest loading rate ($\dot{\delta }$, 2.20 × 10^−3^ mm/s) is set as a quasi-static condition. H is the drop height of the hammer for the impact tests.

**Figure 8:**
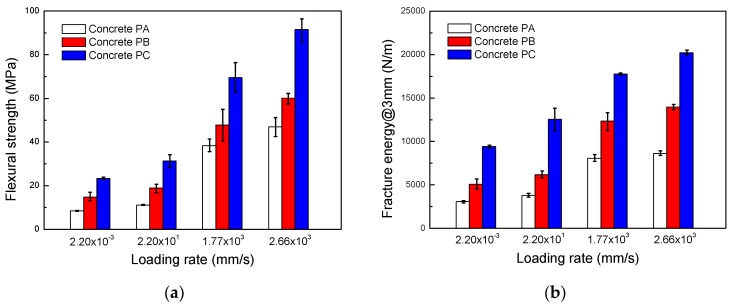
Flexural strength and fracture energy at different loading rates. (**a**) Flexural strength; (**b**) Fracture energy@3mm.

Figure 8 shows the flexural strength and the fracture energy at four loading rates for three different SFRSCCs. Regardless of the loading rates, it is obvious that higher fiber content exhibits a higher fracture energy and flexural strength due to the improvement of the fiber bridging capacity at the crack surface.

**Figure 9:**
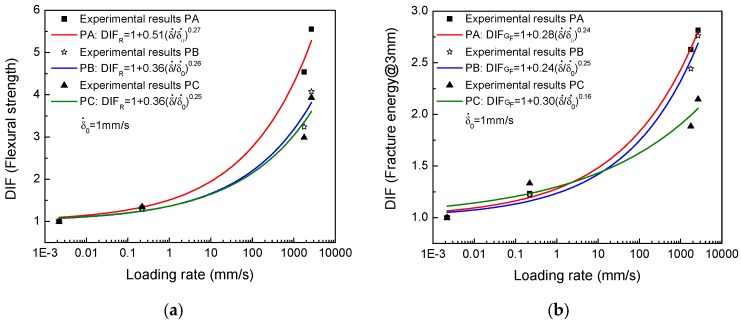
(**a**) Loading rate effect on the flexural strength (**b**) Loading rate effect on the fracture energy@3mm.

The tendency of the rate effect on flexural strength and fracture energy is also presented in Figure 9. It has been observed that flexural strength increases with the increase in loading rates for each type of SFRSCC. It should be noted that the rate effect is minor under low loading rates. For instance, the DIF for the three different concretes is approximately 1.3 at the low loading rate of 2.20 × 10^1^ mm/s, namely, the enhancement of flexural strength is around 30%. However, under impact conditions, the rate effect is remarkable. The DIF ranges from 2.98 to 5.55. Moreover, the high performance SFRSCCs (Concretes PB and PC) are less sensitive to loading rates than conventional SFRSCC (Concrete PA). For instance, at the loading rate of 2.66 × 10^3^ m/s, the DIF of flexural strength for Concrete PA is 5.55, while they are 4.07 and 3.93, respectively, for Concretes PB and PC.

Furthermore, a curve fitting of the DIF for each concrete in regard to the flexural strength is derived from the experimental results as shown in Equations (5)–(7) by using the least-squares method. The correlation coefficient is over 95%. Though the format of such equation was original for plain concrete considering rate effect on mechanical properties [44], it is still useful for SFRSCC.

(5) $$\mathrm{Concrete}\;\mathrm{PA}:{\;\mathrm{DIF}}_{R}=\;1+k{\left(\frac{\dot{\delta }}{\dot{\delta_{0}}}\right)}^{n}=1+0.51{\left(\frac{\dot{\delta }}{\dot{\delta_{0}}}\right)}^{0.27},\;\mathrm{for}\;\dot{\delta }\;\mathrm{in}\;\mathrm{mm}/s\;$$

(6) $$\mathrm{Concrete}\;\mathrm{PB}:{\;\mathrm{DIF}}_{R}=1+k{\left(\frac{\dot{\delta }}{\dot{\delta_{0}}}\right)}^{n}=1+0.36{\left(\frac{\dot{\delta }}{\dot{\delta_{0}}}\right)}^{0.26},\;\mathrm{for}\;\dot{\delta }\;\mathrm{in}\;\mathrm{mm}/s\;$$

(7) $$\mathrm{Concrete}\;\mathrm{PC}:{\;\mathrm{DIF}}_{R}=1+k{\left(\frac{\dot{\delta }}{\dot{\delta_{0}}}\right)}^{n}=1+0.36{\left(\frac{\dot{\delta }}{\dot{\delta_{0}}}\right)}^{0.25},\;\mathrm{for}\;\dot{\delta }\;\mathrm{in}\;\mathrm{mm}/s\;$$

where $\dot{\delta }$ is the loading rate in mm/s, $\dot{\delta_{0}}$ is set as 1 mm/s. Thus, the adjustment parameters k and n do not have units. The equations may be used to predict the rate effect on flexural strength and may also be helpful in performing numerical simulations involving fracture of these concretes.

The loading rate effect on the fracture energy@3mm is also shown in Figure 9b. The tendency is similar to that of the flexural strength, that is, the tendency is moderate under low loading rates, and dramatic under high loading rates. A similar equation for each concrete is also fitted to represent this behavior, see Equations (8)–(10). The correlation coefficient is over 92%.

(8) $$\mathrm{Concrete}\;\mathrm{PA}:{\;\mathrm{DIF}}_{G_{F}}=1+m{\left(\frac{\dot{\delta }}{\dot{\delta_{0}}}\right)}^{r}\;=1+0.28{\left(\frac{\dot{\delta }}{\dot{\delta_{0}}}\right)}^{0.24},\;\mathrm{for}\;\dot{\delta }\;\mathrm{in}\;\mathrm{mm}/s\;$$

(9) $$\mathrm{Concrete}\;\mathrm{PB}:{\;\mathrm{DIF}}_{G_{F}}=1+m{\left(\frac{\dot{\delta }}{\dot{\delta_{0}}}\right)}^{r}\;=1+0.24{\left(\frac{\dot{\delta }}{\dot{\delta_{0}}}\right)}^{0.25},\;\mathrm{for}\;\dot{\delta }\;\mathrm{in}\;\mathrm{mm}/s$$

(10) $$\mathrm{Concrete}\;\mathrm{PC}:{\;\mathrm{DIF}}_{G_{F}}=1+m{\left(\frac{\dot{\delta }}{\dot{\delta_{0}}}\right)}^{r}\;=1+0.30{\left(\frac{\dot{\delta }}{\dot{\delta_{0}}}\right)}^{0.16}\;,\;\mathrm{for}\;\dot{\delta }\;\mathrm{in}\;\mathrm{mm}/s$$

where coefficients m and r are adjusting parameters without units due to the fact that $\dot{\delta_{0}}$ is set as 1 mm/s as previously mentioned. Moreover, the rate effect on the fracture behavior of the SFRSCC is also mild in the low rate range. This could be attributed to viscous effects mainly originating from the presence of free water in voids and porous structures in the matrix [51], and also the weak pullout behavior between the fiber and the matrix [52]. However, under impact loading rates, the rate effect is remarkable. On the one hand, the additional microcracking and the additional resistance to microcracks initiation and growth make fracture propagation more difficult [53,54,55]. On the other hand, steel fibers embedded in concrete matrix support a higher load under impact and the pullout energy is also greater and the rate effect is pronounced [56]. Furthermore, the high performance SFRSCCs (Concretes PC and PB), with higher fiber contents, are less sensitive to loading rates than the conventional SFRSCC (Concrete PA).

#### 3.3.3. Equivalent Strain Rate for Various Loading Rates

In the case of three-point bending tests on notched beams, there is no direct relationship between the loading rate and the strain rate around the notch tip due to the complex stress state of the notch tip. In our previous work [27], a strain gauge was bonded to the notch tip to measure the strain rate at different loading rates. It showed results similar to those provided by Equation (11) [57], which was original for the beam without notch. Table 5 presents an estimation of the corresponding strain rates for various loading rates, which would be helpful for comparison with experimental results in the literature.

(11) $$\dot{\varepsilon }=6\left(D-a\right)\dot{\delta }/s^{2}$$

### 3.4. Comparison with Conventional Vibrated Steel Fiber-Reinforced Concrete (CVSFRC)

Table 6 provides a comparison between the experimental results of a CVSFRC with that presented in this paper. It is noteworthy that the impact tests of the CVSFRC were performed by the same drop-weight impact machine [25]. Taking the fiber content and the quasi-static fracture behavior into account, only the results of Concrete PB are listed in the table. It is obvious that the SFRSCC has higher rate sensitivity than the CVSFRC when they have similar compressive strength and quasi-static flexural behavior. For instance, the DIF for R and G_F_@3 mm of the SFRSCC is 4.07 and 2.76, respectively, at the impact velocity of 2.66 × 10^3^ m/s, versus 3.48 and 2.52 (G_F_@2 mm) for the CVSFRC. Nevertheless, it is still a rough comparison, due to the fact that the rate sensitivity is complicated and related to the matrix, fiber content and shape, and the bond behavior between the matrix and the fibers. In order to achieve a better understanding of the difference of rate sensitivity between SFRSCC and CVSFRC, further studies are necessary.

## 4. Conclusions

The dynamic fracture behavior of SFRSCC is seldom explored in the literature. Thus, this paper investigates the fracture behavior of three different SFRSCCs for a wide range of loading rates. It used a conventional SFRSCC and two high-performance SFRSCCs, with corresponding fiber contents of 0.51%, 0.77% (hybrid fibers) and 1.23% (hybrid fibers), respectively. Moreover, their matrix was kept constant. Furthermore, the loading rates varied from a quasi-static level to a dynamic one. The order of magnitudes was from 10^−3^ to 10^3^ mm/s, corresponding to strain rates from 10^−5^ to 10^1^ s^−1^.

A quasi-static three-point bending test for steel fiber-reinforced concrete is still an appropriate method to obtain an elastic modulus. The relative error of the one measured by cylinders is less than 6%.

For both conventional and the high-performance SFRSCCs, the flexural strength and the fracture energy are rate sensitive. At low loading rates, the rate effect is minor, while it is remarkable at high loading rates. At a low loading rate (2.20 × 10^1^ mm/s), for flexural strength, three different SFRSCCs achieve approximately 30% enhancement. While at high loading rates, the dynamic increase factor for the conventional SFRSCC, is approximately 6 versus 4 for the two different high-performance SFRSCCs. Moreover, with an increase in fiber content the rate sensitivity is less. Regarding the fracture energy, the gain is less than 40% for three different SFRSCCs at low loading rate (2.20 × 10^1^ mm/s), while it is less than 3 at high loading rates.

Under dynamic loading conditions, the post-peak behavior is also influenced by the fiber content, that is, the second peak load or multiple ones increases with fiber content. This improves residual flexural performance.

Two empirical equations for the rate sensitivity of flexural strength and fracture energy are proposed for each type of SFRSCC. They would be helpful in numerical simulations that evaluate the rate effect of the fracture behavior of these concretes.

## Figures and Tables

**Table 1:** Steel fiber contents and results of slump flow tests. Concrete with deflection-softening behavior (Concrete PA); concrete with mild deflection-hardening behavior (Concrete PB); concrete with marked deflection-hardening behavior (Concrete PC).

Concrete Type	Smooth Short Fibers Dramix OL 13/0.20 (kg/m^3^)	Hooked-End Long Fibers Dramix RC 80/30 BP (kg/m^3^)	Fiber Volume Ratio	Values of Slump Flow Tests (mm)
PA	40	-	0.51%	700
PB	40	20	0.77%	665
PC	40	60	1.23%	570

**Table 2:** Properties of concretes at an age around seven months.

Concrete Type	Compressive Strength	Elastic Modulus	Poisson’s Ratio	Density
	fc	E	ν	ρ
	(MPa)	(GPa)		(kg/m^3^)
PA	112 (1)	46.4 (3)	0.18 (0.01)	2362 (3)
PB	112 (6)	45.2 (2)	0.17 (0.01)	2376 (32)
PC	114 (3)	45.9 (3)	0.17 (0.01)	2408 (11)

Note: values in parentheses are standard deviations. Four tests were conducted for each concrete.

**Table 3:** Comparison of elastic modulus.

Concrete Type	Elastic Modulus (GPa)		Relative Error
	From Cylinder	From Beam	
PA	46.4 (3)	44.8 (2)	3.4%
PB	45.2 (2)	42.9 (3)	5.1%
PC	45.9 (3)	44.0 (4)	4.1%

Note: values in parentheses are standard deviations. Four tests were conducted for each concrete.

**Table 4:** Experimental results at different loading rates.

Concrete Type	Testing Machine	H (mm)	$\dot{\delta }\;$(mm/s)	Pmax (kN)	R (MPa)	DIF for R	G_F_@3 mm (N/m)	DIF for G_F_@3 mm
PA Conventional SFRSCC	Servo-hydraulic testing machine	-	2.20 × 10^−3^	11.96(0.5)	8.46(0.3)	1	3067(264)	1
		-	2.20 × 10^1^	15.70(0.4)	11.13(0.4)	1.32	3784(420)	1.23
	Drop-weight impact machine	160	1.77 × 10^3^	55.13(6)	38.41(4)	4.54	8060(774)	2.63
		360	2.66 × 10^3^	67.89(9)	46.98(6)	5.55	8635(547)	2.82
PB High-performance SFRSCC	Servo-hydraulic testing machine	-	2.20 × 10^−3^	20.85(4)	14.74(3)	1	5056(1168)	1
		-	2.20 × 10^1^	26.85(4)	18.93(3)	1.28	6171(789)	1.22
	Drop-weight impact machine	160	1.77 × 10^3^	67.77(15)	47.77(10)	3.24	12347(2078)	2.44
		360	2.66 × 10^3^	85.47(5)	60.07(5)	4.07	13964(609)	2.76
PC High-performance SFRSCC	Servo-hydraulic testing machine	-	2.20 × 10^−3^	33.06(0.8)	23.28(0.05)	1	9414(361)	1
		-	2.20 × 10^1^	43.98(6)	31.28(4)	1.34	12552(2646)	1.33
	Drop-weight impact machine	160	1.77 × 10^3^	98.41(14)	69.53(11)	2.98	17752(230)	1.89
		360	2.66 × 10^3^	129.67(11)	91.50(6)	3.93	20218(588)	2.15

Note: values in parentheses are standard deviations. Four tests were performed at each loading rate. Drop height of the hammer for the impact tests (H); dynamic increase factor (DIF).

**Table 5:** Equivalent strain rate for various loading rates.

Loading Rate (mm/s)	Strain Rate (s^−1^)
2.20 × 10^−3^	1.0 × 10^−5^
2.20 × 10^1^	9.9 × 10^−2^
1.77 × 10^3^	8.1 × 10^0^
2.66 × 10^3^	1.2 × 10^1^

Note: Mean value of 12 specimens at each loading rate.

**Table 6:** Comparison of experimental results.

Dimensions, Compositions and Properties	Conventional Vibrated Steel Fiber Reinforced Concrete (CVSFRC) [25]	Steel Fiber Reinforced Self-Compacting Concrete (SFRSCC)
Beam size: B × D × S * (mm^3^)	150 × 150 × 500	100 × 100 × 333
Notch: a (mm)	25	17
Fiber content (volume ratio)	0.83%	0.77%
Mix proportions: C:S:A:W **	1:1.6:1.2:0.29	1:2.48:0:0.38
Steel fiber shape	Hooked-end	Smooth + hooked-end
l/d ***	50/0.75	13/0.20 + 30/0.38
(Aspect ratio)	(67)	(65 and 80)
fc (MPa)	92	112
E (GPa)	35	45
Quasi-static load-deflection curve	High performance (deflection-hardening)	High performance (deflection-hardening)
DIF for R at 1.77 × 10^3^ m/s	2.71	3.24
DIF for G_F_*@3* mm at 1.77 × 10^3^ m/s	1.83	2.44
DIF for R at 2.66 × 10^3^ m/s	3.48	4.07
DIF for G_F_*@2* mm at 2.66 × 10^3^ m/s	2.52	2.76

* B × D × S, Width × Depth × Span. ** C:S:A:W, Cement:Sand:Aggregate:Water, mix proportions by weight. *** l/d, Length/diameter of the steel fiber.
